# Applying Multivariate Clustering Techniques to Health Data: The 4 Types of Healthcare Utilization in the Paris Metropolitan Area

**DOI:** 10.1371/journal.pone.0115064

**Published:** 2014-12-15

**Authors:** Thomas Lefèvre, Claire Rondet, Isabelle Parizot, Pierre Chauvin

**Affiliations:** 1 Inserm, UMRS 1136, Pierre Louis Institute of Epidemiology and Public Health, Department of Social Epidemiology, Paris, France; 2 Sorbonne Universités, UPMC Univ Paris 06, UMRS 1136, Paris, France; 3 Sorbonne Universités, UPMC Univ Paris 06, Faculty of Medicine Pierre and Marie Curie, Department of general practice, Paris, France; 4 CNRS, UMR 8997, Centre Maurice Halbwachs, Research group on social inequalities, Paris, France; UNC School of Dentistry, University of North Carolina-Chapel Hill, United States of America

## Abstract

**Background:**

Cost containment policies and the need to satisfy patients’ health needs and care expectations provide major challenges to healthcare systems. Identification of homogeneous groups in terms of healthcare utilisation could lead to a better understanding of how to adjust healthcare provision to society and patient needs.

**Methods:**

This study used data from the third wave of the SIRS cohort study, a representative, population-based, socio-epidemiological study set up in 2005 in the Paris metropolitan area, France. The data were analysed using a cross-sectional design. In 2010, 3000 individuals were interviewed in their homes. Non-conventional multivariate clustering techniques were used to determine homogeneous user groups in data. Multinomial models assessed a wide range of potential associations between user characteristics and their pattern of healthcare utilisation.

**Results:**

We identified four distinct patterns of healthcare use. Patterns of consumption and the socio-demographic characteristics of users differed qualitatively and quantitatively between these four profiles. Extensive and intensive use by older, wealthier and unhealthier people contrasted with narrow and parsimonious use by younger, socially deprived people and immigrants. Rare, intermittent use by young healthy men contrasted with regular targeted use by healthy and wealthy women.

**Conclusion:**

The use of an original technique of massive multivariate analysis allowed us to characterise different types of healthcare users, both in terms of resource utilisation and socio-demographic variables. This method would merit replication in different populations and healthcare systems.

## Introduction

In the European context of cost-containment policies and the post-2008 economic and financial crisis [1], cost optimisation and, in some countries, cost reduction of public expenditure has become unavoidable and the healthcare system is no exception. For this reason, the healthcare system may need to be adapted to cost-containment goals while at the same time meeting patients’ needs and expectations as closely as possible. This requires, among other issues, accurate characterisation of healthcare resource utilisation by the user population, as well as identification of determinants of use.

Many studies have previously addressed the use of the healthcare system (either individual services, or globally) by the general population or by specific population subgroups. For example, several studies have examined healthcare system utilisation from a systemic point of view or from a decision-making approach [2]–[4], or by subgroups of the population, such as cancer survivors [5], migrants [6], [7], or the underserved and low-income people [8]–[10]. In addition, determinants of utilisation of specific healthcare services have been investigated, including mental healthcare services [11], emergency care units [12], primary care resources [13], dental care [14] and specialist consultations [15]. Associations between health insurance and healthcare research have also been regularly documented [16].

It has been suggested that healthcare systems themselves could not be analysed through a classical reductionist approach but should be considered as complex systems [17] which require analysis with non-conventional techniques. In particular, it could be interesting to identify distinct groups of patients which would exhibit different homogeneous patterns of resource utilisation. If such groups can be identified, then factors associated with each utilisation profile can be examined using conventional approaches [18]–[20].

Identifying such utilisation patterns requires the use of particular multivariate techniques, which are capable of taking into account a vast amount and variety of variables simultaneously, documented from the largest population possible. These techniques, particularly clustering techniques, have been applied and validated in a wide range of areas of medicine, including genetics [21]–[23], imaging [24]–[26], clinical medicine [27], [28] and public health [29].

In this study, we aimed to identify and characterise distinct profiles of users of the French healthcare system in an urban environment, through analysis of data from a representative, population-based study in the Paris metropolitan area, using clustering techniques.

## Methods

This work is based on the SIRS cohort study that received legal authorization from two French national authorities for non-biomedical research: the Comité consultatif sur le traitement de l’information en matière de recherche dans le domaine de la santé (CCTIRS) and the Commission nationale de l’informatique et des libertés (CNIL) [30]. The participants provide their verbal informed consent. Written consent was not necessary because this survey did not fall into the category of biomedical research (as defined by French law).

This study represents a cross-sectional analysis of data collected in the SIRS cohort study in 2010 among a representative sample of 3,000 French-speaking adults in the Paris metropolitan area (Paris and its suburbs, a region with a population of 6.5 million).

### The SIRS cohort

The SIRS cohort was constituted in 2005 using a 3-level random sampling method. In a first step, 50 census blocks (with about 2000 inhabitants each) were randomly selected using a stratification based on socioeconomic status and whether they qualified or not for “underprivileged urban area” according to the central government list. In the next step, 60 households were randomly chosen from a complete list of households within each selected census block. In the final step, one adult was randomly selected from each household by the birthday method. The refusal rate among the newly contacted people was 29%. The methodology of the SIRS study and detailed characteristics of the study population have been described previously elsewhere, for example in [31].

### Characterisation of healthcare utilisation

A comprehensive, detailed profile of the French healthcare system is provided in reference [32]. Interviewees were asked in detail about their own use of healthcare services during the twelve months preceding the interview. All responses were codes as categorical variables and all reference periods were the last twelve months. Resource use was grouped into categories as detailed below. Unless otherwise specified, all consultation frequencies fell into one of five categories (none, only once, only twice, 3–5 times, or ≥6 times).

**Primary care:** French people seeking healthcare may consult a general practitioner (GP), whether as an end in itself or as an entry point to specialists (the French system has adopted this gate-keeping model since 2004 [32]). Patients need to respect this procedure so that they can be reimbursed. Exceptions are made for four kinds of specialists who can be consulted directly, namely gynaecologists (who are mainly community-based in France), ophthalmologists, paediatricians and psychiatrists; these four specialities will henceforward be referred to as direct access specialists (DAS). We used six variables to characterise primary care utilisation, namely date of the latest dental consultation (4 categories: less than 2 years, between 2 and 3 years, more than 3 years, never), having declared a referring GP (yes/no), frequency of GP consultation, frequency of DAS consultation, undergoing a medical check-up in a dedicated Social security centre (yes/no), frequency of requests for medical advice from friends and relatives (4 categories: none, 1 or 2, 3 to 10, more than 10 times).

**Indirect access to a specialist (IAS):** IAS concerns all other specialists except DAS. The patient may access to them only when referred by their GP (or from their own initiative but at full cost). A single variable was documented, the frequency of IAS consultations.

**Paramedical or alternative care:** two variables were considered: having consulted an acupuncturist or an osteopath (yes/no) and having consulted for non-conventional or alternative healthcare (yes/no). Traditional Chinese medicine fell into the latter category.

**Site of healthcare consumption:** in France, healthcare can be delivered in three principal settings: public hospitals or clinics, private hospitals or clinics, and community settings. Since the place of consultation was systematically documented for each medical consultation over the previous twelve months, six distinct variables were considered: having consulted (at least once) a GP in a public hospital or clinic, a private hospital or clinic, or in a community setting, and having consulted (at least once) a specialist in a public hospital or clinic, a private hospital or clinic, or in a community setting.

**Emergency care:** two variables documented healthcare utilisation in emergency situations, depending on the place where care was delivered; these were the frequency of home visits for emergency reasons and the frequency of consultations in an emergency unit.

### Population characteristics and factors associated with healthcare utilisation

Five dimensions were explored as possibly associated with healthcare utilisation, all of them made up of three items (except for the socioeconomic status, with four items).

**Demographics:** age (5 categories: 18–29, 30–44, 45–59, 60–74, and 75 years old or more), gender, origin (distinguishing, as previously reported [33], [34], between French people born to two French parents, French people born to at least one foreign parent, and foreign immigrants).

**Socioeconomic status:** education level (none or primary/secondary/tertiary), employment status (employed, unemployed, inactive or retired), monthly household income per consumption unit (in quintiles, and computed as the total household income divided by the number of consumption units [adult: 1; child ≥14 years: 0.5; child <14 years: 0.3]), according to the usual OECD-modified scale recommended by Eurostat, and health insurance status (full coverage by the statutory health insurance – SHI, SHI plus a voluntary health insurance - VHI, full coverage by a special insurance for the poor, partial coverage by the SHI only, and no insurance at all).

**Stance regarding health and medicine:** general attitude toward medical consultation (people were asked if they generally consult a doctor as a last resort, or as soon as they are not feeling well), having a relative or a friend suffering from a severe condition, and having medical professionals among relatives.

**Social integration:** feeling of isolation (very isolated, rather isolated, rather supported, very supported), level of social support (low, medium, high) and frequency of social contacts (quartiles), both as described in [34].

**Perceived health:** as measured by the Minimum European Health Module [35], [36] that assembles the global perceived health status (good, average, bad), the global activity limitation indicator (presence of a long-standing activity limitation in the previous six months), and the presence of a chronic or long standing health problems over the twelve months.

These dimensions are similar to those identified by Anderson in the late 60’s [37], [38]. In his Behavioral Model of Health Service Use (BMHSU), this author distinguished between three classes of factors: predisposing factors (such as age, gender, education, occupation, social relationships, attitudes and knowledge related to health services and professionals), enabling factors (such as income or health insurance), and need factors (such as perceived health status or functional disability). According to this model, Andersen suggested that the respective roles of these factors may provide clues for measuring equity in service use. For example, if the main drivers of health service use are need factors, access can be considered equitable. Conversely, if the main drivers are constituted of social factors, beliefs and enabling factors, access can be considered as not equitable.

### Clustering methods

The use of clustering techniques available for health scientists has been described previously [39]–[41]. Clustering techniques require the determination of some data-specific parameters, such as the number of groups to be retrieved. In order to identify the different types of healthcare system utilisation, we used the partitioning around medoïds (PAM) algorithm with the Euclidean distance as a reference analysis and applied it to the healthcare system utilisation variables [40]. A resampling-based scheme and cluster-robustness approach [42] was used to determine the key parameters of the algorithm and in particular the number of clusters. Other clustering methods or sets of parameters were further used for sensitivity analyses. The PAM algorithm was run with alternative distance or similarity measure (Manhattan distance and Gower measure [40]). A fuzzy-logic version of PAM, the FANNY algorithm [40], was also applied to the data, with several values for the fuzziness parameter. All analysis was conducted on R 2.13.1 (R Foundation for Statistical Computing, 2012), with the clusterCons package.

### Statistical analyses

We accounted for the three-level sampling design of the SIRS cohort by using the *survey* command options of the STATA IC 10 software (STATA Corp, 2007) for descriptive statistics and multinomial models. Classical ratio tests (chi-square or exact Fisher) were used to compare population characteristics according to their type of care utilisation.

We used multinomial regression models to investigate significant associations between variables of each of the five dimensions studied, and the types of healthcare utilisation as categorical outcomes. Adjusted odd ratios (OR) are reported with a *p* value for linear trend. Statistical significance was assessed at a bilateral *p* value<0.05.

## Results

### Cluster identification

**Figure 1:**
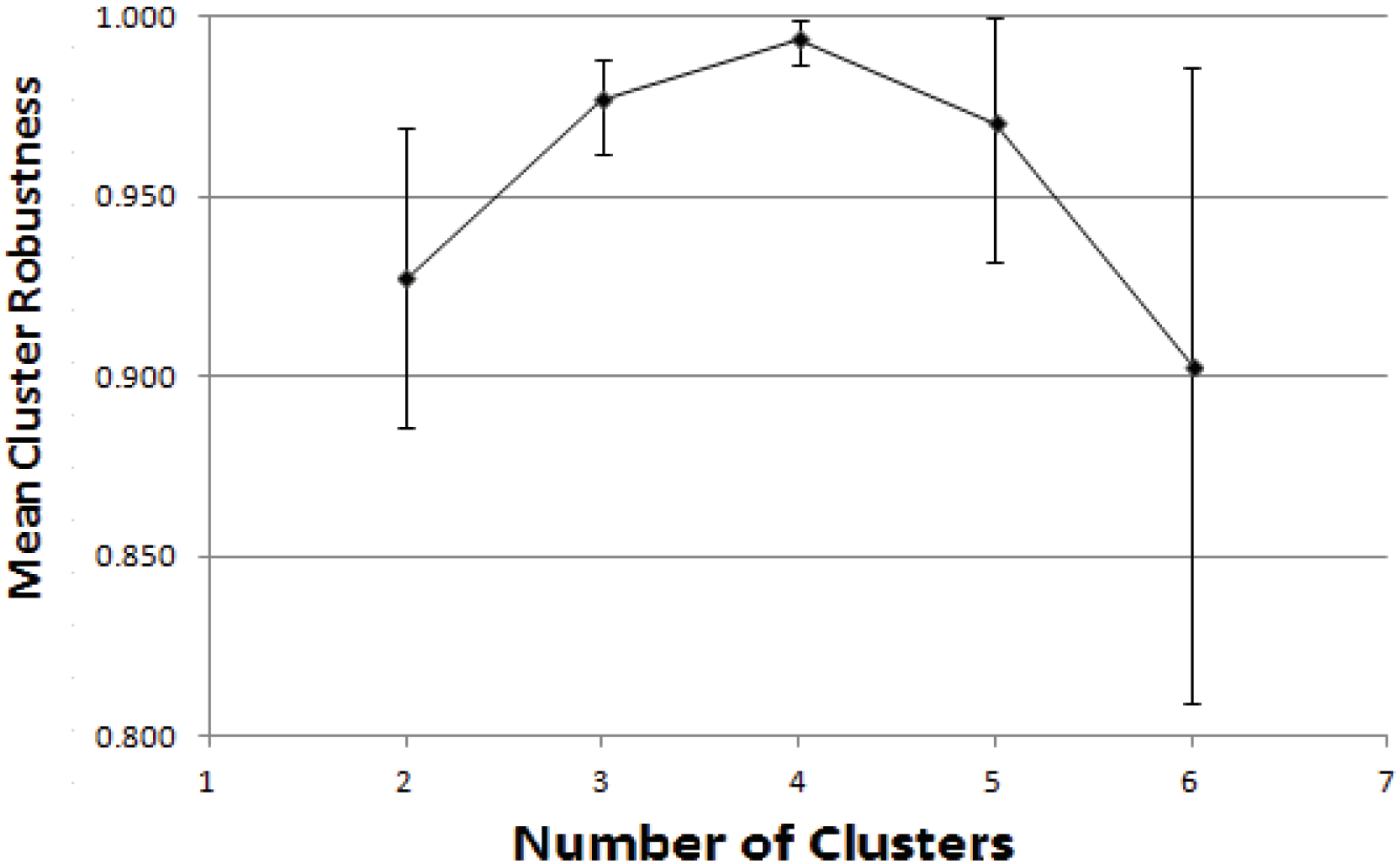
Cluster robustness according to the number of assumed clusters in the data. Mean cluster robustness, together with minimum and maximum values, is presented as a function of the number of searched clusters.

The optimal number of individual clusters that would account for the data was four. This was the value at which mean cluster robustness was maximal and the range of robustness values narrowest (Fig. 1). A cluster robustness of unity indicates that no member of a given cluster was likely to be assigned to another cluster when the algorithm was reiterated and also directly reflects the stability of the cluster. In our analysis, mean robustness for the four-cluster model was high >0.99, indicating that very few individuals could not be assigned unequivocally to a given cluster.

### Sensitivity analyses

No qualitative differences in cluster distribution or robustness were identified by reiterating the algorithm with alternative distance or similarity measures. Qualitatitively identical findings were obtained in all models (results not shown).

### The four types of healthcare utilisation

The four clusters identified in our data were associated with four distinct types of healthcare utilisation, accounting for 30.0% (Type 1), 21.0% (Type 2), 25.7% (Type 3) and 23.3% (Type 4) of the study population. Table 1 shows the contribution of each variable of healthcare utilisation to each Type.

**Table 1:** Resource utilisation by the four types of healthcare utilisation in the Paris metropolitan area, 2010.

	Type 1	Type 2	Type 3	Type 4	All types		p
	(30.0%)	(21.0%)	(25.7%)	(23.3%)	N = 3006		
**Primary care**							
Date of the last dentist consultation							<0.001
<2 yr	84.3	68.1	72.9	87.0	78.2		
2–3 yr	4.0	9.8	9.2	4.3	6.8		
>3 yr	7.8	16.8	13.1	6.2	10.9		
never	3.9	5.3	4.8	2.5	4.1		
Having a referring GP							<0.001
	96.0	71.5	93.2	90.6	88.1		
Frequency of consultationwith a GP							<0.001
0	2.4	60.4	-	6.7	16.9		
1	9.0	39.6	-	23.7	17.7		
2	17.0	-	31.8	23.9	18.1		
3–5	38.6	-	47.9	34.6	30.5		
6+	33.0	-	20.3	11.1	16.8		
Frequency of consultation witha DAS							<0.001
0	49.3	87.7	93.6	-	57.8		
1	22.3	12.2	6.4	40.2	20.2		
2	11.4	0.1	-	25.1	9.1		
3–5	9.4	-	-	16.3	6.5		
6+	7.6	-	-	18.3	6.4		
Has had a medical check-up ina dedicated centre							0.127
	7.0	5.1	5.7	3.6	5.4		
Frequency of requests for medicaladvice from relatives							0.0057
0	78.1	85.3	84.4	78.3	81.4		
1–2	14.2	10.7	12.3	14.7	13.0		
3–10	6.1	3.8	3.4	6.3	4.9		
11+	1.6	0.2	-	0.7	0.6		
**Paramedical and alternative care**							
Has consulted anacupuncturist/osteopath						<0.001	
	22.7	6.4	9.4	17.1	17.1		
Has consulted fornon-conventional/alternativehealthcare						<0.001	
	7.2	2.1	3.3	6.9	4.9		
**Indirect access specialists**							
Frequency of consultationwith an IAS						<0.001	
0	-	81.1	78.4	68.0	54.8		
1	1.3	12.2	21.6	28.6	15.3		
2	29.3	1.8	-	3.3	9.4		
3–5	40.8	-	-	0.1	12.6		
6+	28.7	-	-	-	0.8		
**Site of healthcare consumption**							
Public hospital or clinic-GP	12.3	3.1	14.1	7.6	9.4	<0.001	
Public hospital or clinic-specialist	42.5	5.5	7.2	19.9	19.6	<0.001	
Private hospital or clinic-GP	14.2	1.8	10.5	6.3	8.4	<0.001	
Private hospital or clinic-specialist	21.1	1.5	3.5	9.3	9.3	<0.001	
Ambulatory settings-GP	91.0	34.9	90.9	88.6	76.8	<0.001	
Ambulatory settings-specialist	83.0	22.3	16.3	88.8	53.4	<0.001	
**Emergency care**							
Frequency of home visits						0.002	
0	-	81.1	78.4	68.0	54.8		
1	1.3	12.2	21.6	28.6	15.3		
2	29.3	1.8	-	3.3	9.4		
3–5			40.8	-	-	0.1	
6+	28.7	-	-	-	0.8		
Frequency of consultations inemergency units						<0.001	
0	77.5	89.8	80.7	83.0	82.6		
1	15.9	9.7	15.7	13.0	13.7		
2	4.0	0.1	2.2	2.1	2.3		
3–5	2.2	-	1.2	1.6	1.3		
6+	0.4	-	0.2	0.3	0.2		

GP: General Practitioner. DAS: Direct Access Specialist. IAS: Indirect Access Specialist. All results are expressed in percentages.

Type 1 represents the largest users of primary care. These individuals used all available resources, including GPs, social security centres for check-ups and medical advice from relatives, and used these resources extensively. For example, 71.6% had consulted their GP three times or more in the past twelve months and also consulted DAS extensively (50.7% with at least one visit). They were also the most frequent users of IAS, with 100% having consulted at least one IAS in the last 12 months and 69.5% consulted more than two times. They were also the largest users of paramedical or alternative care. Type 1 individuals consulted in all settings (principally in public hospitals for specialists and in community care for GPs) and were the largest users of the private sector. Finally, they were also the principal users of emergency care, both in the home (12.9%) and in emergency units (22.5%).

Type 2 was the mirror image of Type 1. Together with Type 3, individuals in Type 2 were the least frequent users of primary care. In Type 2, 28.5% of individuals had no referring GP and only 12.3% had consulted a DAS (furthermore, only once in most cases). Although 18.9% had consulted an IAS in the previous year, but only 4.9% consulted more than twice. These individuals rarely used paramedical or alternative resources. Whatever the setting, Type 2 users had the lowest rate of healthcare utilisation, and this was especially true for private hospitals and clinics. Type 2 individuals rarely required emergency care in the home (3.5%) or in emergency units (9.8%) and, when they did, they usually (9.7%) consulted only once and hardly ever more than twice (0.1%).

Healthcare resource utilisation by Type 3 users was closer to that observed in Type 2 than that in Types 1 or 4. Type 3 individuals were characterised by extensive recourse to GPs, with 20.3% of users have consulted more than six times in the last twelve months. In contrast, they rarely consulted DAS (6.4%) or IAS (only 21.6% of them had consulted an IAS and never more than once). They seldom used paramedical or alternative care (9.4% and 3.3% respectively). When consulting GPs, they were more likely to consult in community care, and had little use for the public sector. Nonetheless, Type 3 users constituted the second most frequent user of emergency resources, especially in emergency units (19.3% consulted at least once in emergency units).

Type 4 shared similarities with Type 1, in that it was constituted by people who were heavy users of the healthcare system. Type 4 did not present a particularly high rate of GP consultations (being the third-most frequent user) but had the highest use of DAS (100%). Type 4 individuals were also the second most frequent users who referred to relatives for medical advice, and who consulted for paramedical or alternative care. Type 4 was also the second highest user of IAS, although the frequency of consultation was relatively low (21.6% had consulted once and none consulted more than once). Individuals in Type 4 used all settings when consulting specialists (public and private hospitals, as well as community care). For emergency care, Type 4 presented the lowest level of use compared to the other Types, with 17.0% having consulted in emergency units, 13.0% only once, and 1.6% between three and five times (second user in that case).

### Factors associated with healthcare utilization

Univariate associations between independent variables and each of the four profiles of healthcare utilisation are presented in Table 2. Only three variables were not associated with significant differences between profiles, namely having medical professionals among relatives, feelings of isolation and frequency of social contacts.

**Table 2:** Population characteristics of the four types of healthcare utilisation in the Paris metropolitan area, 2010.

	Type 1	Type 2	Type 3	Type 4	All types	p
	(30.0%)	(21.0%)	(25.7%)	(23.3%)	N = 3006	
**Model 1: Demographics**						
Age (yr.)						<0.001
18–29	11.6	32.1	21.2	23.7	21.8	
30–44	22.8	32.0	35.0	39.5	31.9	
45–59	25.5	23.4	20.2	23.7	23.3	
60–74	24.2	9.3	14.1	9.6	14.7	
75+	15.9	3.3	9.5	3.5	8.3	
Gender						<0.001
Male	42.6	64.3	59.6	21.0	47.0	
Female	57.4	35.7	40.4	79.0	53.0	
Origin						0.006
French, born to Frenchparents						
	72.2	63.3	61.1	69.1	66.6	
French, born to at leastone foreign parent						
	18.3	22.0	23.2	20.1	20.8	
Foreigner	9.5	14.7	15.7	10.8	12.6	
**Model 2: Socioeconomic status**						
Education level						<0.001
Tertiary	59.3	54.5	48.7	63.1	56.5	
Secondary	32.5	38.0	42.3	32.2	36.2	
Primary or none	8.2	7.5	9.0	4.7	7.3	
Employment status						<0.001
Employed	47.9	61.6	55.5	63.1	56.7	
Unemployed	5.7	8.6	9.3	7.1	7.6	
Inactive	46.4	29.8	35.2	29.8	35.7	
Income (quintiles)						
1^st^	16.5	22.8	24.6	18.9	20.6	<0.001
2^nd^	14.2	22.4	23.0	18.5	19.3	
3^rd^	22.3	21.8	19.7	23.2	21.7	
4^th^	20.0	18.2	15.6	17.8	18.0	
5^th^	27.0	14.8	17.1	21.6	20.4	
Health insurance status						<0.001
SHI+VHI	94.1	78.5	85.3	89.6	87.1	
special insurance for the poor	1.7	4.1	2.1	3.0	2.6	
SHI only	3.6	16.6	12.4	6.8	9.7	
None	0.6	0.8	0.2	0.6	0.6	
**Model 3: Stance regarding** **health and medicine**						
General attitude toward medicalconsultation						<0.001
As a last resort	33.0	68.7	38.7	43.2	45.4	
As soon as not feeling well						
	67.0	31.3	61.3	56.8	54.6	
Having a relative or a friendsuffering from a severe condition						0.002
No	51.5	42.7	41.8	53.0	47.3	
Yes	48.5	57.3	58.2	47	52.3	
Having medical professionalsamong relatives						0.215
No	55.7	59.3	61.8	55.8	58.1	
Yes	44.3	40.7	38.2	44.2	44.2	
**Model 4: Social integration**						
Isolation feeling						0.251
Very supported	29.7	35.2	32.0	34.5	32.7	
Rather supported	55.6	54.1	53.7	52.5	54.1	
Rather isolated	12.5	9.6	12.1	12.3	11.6	
Very isolated	2.2	0.1	0.2	0.7	1.6	
Level of social support						<0.001
High	81.9	90.7	87.4	91.3	87.6	
Medium	13.8	6.5	9.1	6.2	9.1	
Low	4.3	2.8	3.5	2.5	3.3	
Frequency of social contacts(quartiles)						0.054
1^st^	25.0	23.3	22.5	18.7	22.5	
2^nd^	24.2	24.9	27.1	21.1	24.4	
3^rd^	26.3	21.6	22.0	30.4	25.1	
4^th^	24.5	30.2	28.4	29.7	28.0	
**Model 5: Health status**						
Perceived health status						<0.001
Good	62.3	92.2	77.4	82.4	78.0	
Average	28.5	6.8	20.0	15.2	18.0	
Bad	9.2	1.0	2.6	2.4	4.0	
Chronic or long standinghealth problem						<0.001
No	40.2	86.4	64.4	72.2	64.8	
Yes	59.8	13.6	35.6	27.8	35.2	
Long standing activitylimitation						<0.001
No	63.1	94.4	81.1	86.5	80.6	
Yes	36.9	5.6	18.9	13.5	19.4	

All results are expressed in percentages.

The five multivariate multinomial models are successively presented in Table 3, with Type 4 being considered as the reference type. As in the univariate analysis, many significant associations were observed. For instance, the probability of belonging to Type 1 increased with age, and women were most likely to belong to Type 4. Foreigners was more likely to belong to Type 3 (OR = 1.95, 95% CI =  [1.30–2.93]), as did individuals with a low education level (primary school or none; OR = 2.26, 95% CI =  [1.39–3.69]). Inactive or wealthy people were most likely to belong to Type 1 (OR = 2.08, 95% CI =  [1.59–2.71], and OR = 1.80, 95% CI =  [1.20–2.70], respectively). Individuals with a SHI only had the highest probability of belonging to Type 2. Referring to their GP for the slightest health issue was an attitude associated with Type 1 (OR = 1.55, 95% CI =  [1.19–2.01]), while the opposite attitude was associated with Type 2. Among the three variables related to social integration, only the frequency of social contacts tended to be associated with the type of healthcare utilisation; although the association was not significant, the point estimate indicated that frequent social contacts were more characteristic of Type 4 people. In terms of health status, reporting a chronic condition was significantly associated with Types 1 and 3 (OR = 2.91, 95% CI =  [2.27–3.71], and OR = 1.34, 95% CI =  [1.02–1.76], respectively), while reporting a good health status tended to be more frequent in Type 2 (OR = 0.53, CI =  [0.33–0.84]).

**Table 3:** Characteristics associated with the type of healthcare utilisation: multinomial logistic regression model (with Type 4 as reference), Paris metropolitan area, 2010.

	Type 1	Type 2	Type 3	p*
	OR [CI 95%]	OR [CI 95%]	OR [CI 95%]	
**Model 1: Demographics**				
Age (yr.)				<0.001
18–29	Ref	Ref	Ref	
30–44	1.18 [0.72–1.93]	0.60 [0.37–0.96]	0.99 [0.67–1.45]	
45–59	2.24 [1.35–3.73]	0.77 [0.48–1.31]	1.01 [0.67–1.55]	
60–74	5.44 [3.28–9.03]	0.80 [0.48–1.31]	1.87 [1.17–2.97]	
75+	11.14 [5.67–21.89]	1.00 [0.47–2.12]	4.45 [2.49–7.94]	
Gender				<0.001
Male	Ref	Ref	Ref	
Female	0.32 [0.22–0.46]	0.14 [0.11–0.20]	0.17 [0.11–0.24]	
Origin				0.0375
French, born to Frenchparents				
	Ref	Ref	Ref	
French, born to at leastone foreign parent				
	1.10 [0.79–1.54]	1.32 [0.89–1.95]	1.54 [1.11–2.17]	
Foreigner	1.19 [0.72–1.99]	1.67 [1.06–2.61]	1.95 [1.30–2.93]	
**Model 2: Socioeconomic status**				
Educational level				0.0119
Tertiary	Ref	Ref	Ref	
Secondary	1.23 [0.89–1.69]	1.25 [0.91–1.71]	1.61 [1.24–2.10]	
Primary or none	1.94 [1.09–3.48]	1.67 [0.92–3.01]	2.26 [1.39–3.69]	
Employment status				<0.001
Employed	Ref	Ref	Ref	
Unemployed	1.40 [0.65–3.00]	0.87 [0.43–1.76]	1.20 [0.61–3.36]	
Inactive	2.08 [1.59–2.71]	1.01 [0.77–1.34]	1.25 [0.98–1.61]	
Income per consumptionunit (quintiles)				<0.001
1^st^	Ref	Ref	Ref	
2^nd^	0.96 [0.61–1.51]	1.11 [0.70–1.76]	1.05 [0.68–1.61]	
3^rd^	1.39 [0.87–2.20]	0.92 [0.53–1.60]	0.82 [0.51–1.29]	
4^th^	1.63 [0.96–2.77]	1.11 [0.62–1.99]	0.91 [0.57–1.47]	
5^th^	1.80 [1.20–2.70]	0.79 [0.51–1.21]	0.90 [0.56–1.43]	
Health insurance status				0.0058
SHI+VHI	Ref	Ref	Ref	
special insurance for thepoor				
	0.55 [0.24–1.22]	1.45 [0.69–3.05]	0.59 [0.26–1.34]	
SHI only	0.63 [0.29–1.37]	2.72 [1.39–5.35]	1.78 [0.89–3.56]	
None	1.01 [0.19–5.41]	1.60 [0.41–6.32]	0.28 [0.04–2.03]	
**Model 3: Stance regarding health and medicine**				
General attitude towardmedical consultation				<0.001
As a last resort	Ref	Ref	Ref	
As soon as not feeling well				
	1.55 [1.19–2.01]	0.33 [0.23–0.47]	1.15 [0.84–1.58]	
Having a relative or afriend suffering from asevere condition				0.0058
No	Ref	Ref	Ref	
Yes	0.97 [0.71–1.32]	0.62 [0.44–0.85]	0.67 [0.48–0.90]	
Having medical professionalsamong relatives				0.3301
No	0.96 [0.77–1.20]	1.19 [0.90–1.59]	1.20 [0.94–1.55]	
Yes	Ref	Ref	Ref	
**Model 4: Social integration**				
Isolation feeling				0.1295
Very supported	Ref	Ref	Ref	
Rather supported	1.11 [0.81–1.52]	0.94 [0.69–1.29]	1.01 [0.72–1.41]	
Rather isolated	0.88 [0.60–1.31]	0.68 [0.43–1.09]	0.90 [0.66–1.22]	
Very isolated	2.84 [1.08–7.50]	1.45 [0.46–4.58]	3.06 [1.06–8.84]	
Level of social support				0.0384
High	Ref	Ref	Ref	
Medium	2.32 [1.40–3.84]	1.03 [0.62–1.72]	1.43 [0.90–2.28]	
Low	1.72 [0.84–3.56]	1.11 [0.59–2.11]	1.28 [0.66–2.49]	
Frequency of socialcontacts (quartiles)				0.0370
1^st^	1.42 [0.96–2.13]	1.28 [0.87–1.90]	1.21 [0.80–1.82]	
2^nd^	1.29 [0.90–1.87]	1.20 [0.87–1.64]	1.32 [0.92–1.89]	
3^rd^	1.03 [0.70–1.53]	0.71 [0.51–1.00]	0.75 [0.46–1.22]	
4^th^	Ref	Ref	Ref	
**Model 5: Health status**				
Perceived health status				<0.001
Good	Ref	Ref	Ref	
Average	1.40 [0.97–2.03]	0.53 [0.33–0.84]	1.18 [0.84–1.67]	
Bad	1.65 [0.71–3.84]	0.75 [0.24–2.36]	0.78 [0.33–1.87]	
Chronic or long standinghealth problem				<0.001
No	Ref	Ref	Ref	
Yes	2.91 [2.27–3.71]	0.48 [0.34–0.67]	1.34 [1.02–1.76]	
Long standing activitylimitation				<0.001
No	Ref	Ref	Ref	
Yes	2.24 [1.44–3.48]	0.56 [0.36–0.88]	1.34 [0.90–2.00]	

*p value for overall trend. Type 4 is the reference type for the estimation of all the Odd-Ratios.

## Discussion

In this study, we took advantage of a database which was representative of the general population of French-speaking adults in the Paris metropolitan area. Because data were recorded from face-to-face interviews, independently from medical registers or medical consumption records, our sample has the advantage of taking into account non-users of healthcare. Social and subjective variables are particularly richly documented in the SIRS cohort, which was originally designed to study social inequalities in health and access to healthcare. We used an original and methodologically robust approach to identify homogenous and consistent types of healthcare system users.

We identified four different types of healthcare user through this approach. The findings of the cluster analysis exhibited strong robustness in terms of sensitivity to parameter tuning and of group stability. One type of user (Type 1) typically consisted of elderly individuals of French origin, who were wealthy but unhealthy, inactive and socially isolated, and who benefited from a good health insurance and took advantage of all kinds of healthcare services, which they used extensively. Type 4 was typically constituted by young, working women of French origin, with a high educational level, who tended to be wealthy and healthy, socially integrated and supported and fully insured. These users were the most likely to frequently consult specialists in the community and to make use of non-conventional care. A third type (Type 2) was constituted by young men, frequently foreigners, who tended to be unemployed and rather poor, healthy but with a mediocre access to health insurance, and who had the lowest utilisation of healthcare services. The last type (Type 3) was constituted of a population of diverse ages, often foreigners, with a poor educational level and low incomes. These users were typically inactive, with a mediocre health insurance, rather socially isolated and unhealthy, and principally used GP services or emergency healthcare.

Our study has certain limitations. Firstly, we dealt with declarative data, without any linkage to medical records or objective measures, so we were unable to estimate possible reporting and recall biases. It is also specific to the French healthcare system and we can thus make no direct comparison with or extrapolate to healthcare systems of other countries, each of which has specific regulation policies (especially in terms of gate-keeping, extent of public and supplementary health insurance and out-of-pocket payments) and provision of healthcare services. For example, if individuals with Type 4 profile present a higher use of specialists, it is partly both because most of them are women and consult a gynaecologist every year. In France, gynaecologists represent general “women’s health” doctors, responsible for all aspects of gynaecological follow-up (including contraception and cervical smears) and who are directly accessible without going through a GP. The place of the gynaecologist in the French healthcare system is atypical and not found in many other countries. Also, despite the existence of a universal basic health insurance, income and insurance status may still influence access to healthcare, which could account for the Type 3 profile. Apart from individuals with the lowest income levels and those suffering from costly chronic diseases, approximately 30% of health expenses are supported by patients (co-payment). They may be reimbursed by a voluntary (supplementary) insurance; but sometimes only partially, according to their contract. In many situations, people also have to pay upfront and are then reimbursed by the basic public health insurance. The gate-keeping system can be bypassed, although this incurs a higher cost or lower reimbursement for patients. In addition, access to IAS and prescription of paraclinical tests such as imaging or laboratory analyses can be prescribed without consulting a GP during an emergency unit consultation instead (with the possibility to get them at the same time rather than in a second step after the GP consultation).

Moreover, even in France, the Paris metropolitan area region is not representative of the whole country, being very urbanised, more wealthy on average and with a higher density of medical provision than the rest of the country, but also with much more social inequalities and spatial segregation than other French regions [43].

Technically speaking, the robustness and stability of the four clusters could result, at least in part, from too many constraints in the analysis methods. In other words, the identified clusters may grossly reflect reality, but lack accuracy. If so, this is likely to be linked to the intrinsic geometric assumptions underlying the clustering methods we used. The overall shape of the groups identified cannot represent complicated configurations, such as reticulated patterns, and the clusters generated by the model are spheroid with little scope for interpenetration. Moreover, we may have encountered a lack of statistical power for some underrepresented categories such as the utilisation of emergency resources.

From the health inequality point of view, our results help clarify some of the differences in behaviours with respect to healthcare system use and to opportunities for healthcare. While it is usually assumed that social inequalities in access to healthcare mainly stem from economic inequalities [44]–[46], it would have been expected that the introduction of universal health insurance in France in 1999 should have removed such barriers [47]. However, it is clear that this has not happened systematically [16], [43], [48]. In fact, the social causes and processes underlying inequalities in access to healthcare are complex, going beyond purely economical or materialistic factors, and involving different psychosocial and behavioural factors as well [49], [50]. Our study may help unravel some of this complexity and diversity. Indeed, we observed several associations between the type of healthcare resource utilisation and social factors previously found to be associated with healthcare indicators, such as objective or perceived health status [45], [51] or, more broadly, health expectations and perceived needs [52], social capital [53] or social integration [54]. These determinants coexist, but contribute to different extents to the four types of utilisation. For example, health status, measured by chronic diseases and functional limitations, was indeed associated with greater use of the healthcare system, together with higher educational level and higher income. Stance regarding healthcare was also found to influence extent of use of the healthcare system and established differences regarding gender and healthcare use were observed. Among all the variables evaluated, only having medical professionals among relatives was not significantly associated with profiles; this may be explained by this question being too general, as it could be interpreted in a wide variety of different ways with respect to closeness, confidence, availability and the professional skills involved. With respect to social integration, the frequency of social contacts appeared to be poorly discriminant. In this context, we believe that the “crude” frequency of social contacts, without any further details on their frequency, quality, context or content, is less meaningful than direct interrogation of global and subjective feelings of isolation. For the latter, significant differences were found for people reporting a “very isolated” status in Types 1 and 3 (Table 3). Taken together, these results, which are consistent with the literature, provide some face validity of our typology.

As mentioned above, this typology can be interpreted according to Andersen’s BMHSU, in terms of equity in access to healthcare services. When looking at the variables that discriminated the four types of user best (OR≥2 or ≤0.5), we observed that the most important predisposing factors for the pattern of healthcare utilisation were age, gender, origins, educational level, a feeling of social isolation and the general attitude toward medical consultation (Types 2 and 4 only), whereas the most prominent enabling factors were the feeling of social isolation and health insurance (Types 2 and 4 only).

Individuals corresponding to the Type 1 profile need to have access to services due to a higher prevalence of chronic conditions and functional limitations, and they also use these services, both because they can afford to (they have adequate financial resources and more time to access services) and because they present habits and perceptions that make them prone to use them. Our study provides no information on whether their access to healthcare meet all their needs, but their pattern of healthcare use does not seem to be explained in terms of predisposing factors such as gender or educational level. Individuals corresponding to the Type 2 profile, who are in majority young males, have a low level of healthcare utilisation which reflects their low perceived needs. At the same time, these individuals are those with the highest proportion of basic insurance status only. This is an obstacle to access to healthcare which is not very reassuring regarding equity in access to health services, particularly since young people may underestimate their real health needs. Individuals corresponding to the Type 3 profile present low rates of use and multiple negative predisposing factors. For example, they are more likely to be foreigners, with a lower educational level and low financial resources. On the other hand, they are likely to suffer from chronic conditions and functional limitations. It is for this profile that the healthcare system is the most likely to be inequitable. Indeed, these individuals predominantly use services from GPs and emergency units, and far less often from specialists. Individuals corresponding to the Type 4 profile have few expressed needs but use services, especially from GPs and DAS, intensively, preferring community care. These individuals have the adequate resources to use services, both economically (income, health insurance) and culturally (female gender, higher educational level, higher social support). In conclusion, of the four profiles described, one (Type 3) and possibly two (Type 2) profiles are in a situation where the French healthcare (and insurance) system is the most likely to be inequitable.

Finally, we demonstrated that the method used was able to reveal stable and meaningful structures in our data without resorting to the usual reductionism of classical studies on healthcare utilisation. For this reason, we think that a similar multivariate clustering method would merit replication in other datasets derived from other contexts, such as non-urban populations or countries with other healthcare systems, in order to confirm or refine our findings.
